# Collaborating With Children and Young People: A New Model for Co-Production

**DOI:** 10.1192/bjo.2022.335

**Published:** 2022-06-20

**Authors:** Aarthi Ravishankar, James Biggin-Lamming, Tom Holliday, Lauren Fraser

**Affiliations:** London North West University Healthcare NHS Trust, London, United Kingdom

## Abstract

**Aims:**

Childhood and adolescence is a time in which the patterns and foundations for future health are laid. The World Health Organisation advocate for providing opportunities for children and young people (CYP) to meaningfully participate in the design and delivery of services. Co-production, in which professionals and citizens collaborate together in an equal partnership, is recommended as an approach to achieve this and is linked to better community relations. Few co-production models exist that are specific to CYP and address the relevant practical and ethical challenges. We propose a new framework which can be used by organisations wishing to engage in meaningful collaboration with CYP.

To create a model for co-production with consideration of the specific needs of CYP.

**Methods:**

The following methodology was used:

- Identification of common themes from ten existing co-production frameworks
- Detailed analysis of three co-production frameworks with reference to CYP
- Identification of key issues from critique of the literature

**Results:**

The key themes incorporated into the model using the above methodology were as follows: Purpose, Assets, Capabilities, Reciprocity, Networks and Relationships, Power, Catalysts, Diversity and Inclusion and Safety and Protection. This co-production framework can be used by organisations that wish to meaningfully collaborate with CYP and assess the depth of co-production of their initiatives.

**Conclusion:**

The new model takes into account the socio-cultural challenges that must be considered when co-producing with CYP including power relations, safety and diversity and inclusion. We advocate for the model being tested, validated and further developed ideally with the collaboration of CYP.

